# Optimizing mycobacteria molecular diagnostics: No decontamination! Human DNA depletion? Greener storage at 4 °C!

**DOI:** 10.3389/fmicb.2023.1104752

**Published:** 2023-04-11

**Authors:** Prajwal Prajwal, Turlough Neary, Katja Rohrbach, Pascal Bittel, Pauline C. Göller, Thorsten Buch, Sebastian Dümcke, Peter M. Keller

**Affiliations:** ^1^Institute for Infectious Diseases, University of Bern, Bern, Switzerland; ^2^Institute of Laboratory Animal Science, University of Zurich, Zurich, Switzerland; ^3^Clemedi AG, Schlieren, Switzerland; ^4^Institute of Social and Preventive Medicine, University of Bern, Bern, Switzerland

**Keywords:** sputum decontamination, NALC-NaOH, saponin, human DNA depletion, molecular diagnostics, mycobacteria, tuberculosis

## Abstract

**Introduction:**

Tuberculosis (TB) is an infectious disease caused by the group of bacterial pathogens *Mycobacterium tuberculosis* complex (MTBC) and is one of the leading causes of death worldwide. Timely diagnosis and treatment of drug-resistant TB is a key pillar of WHO’s strategy to combat global TB. The time required to carry out drug susceptibility testing (DST) for MTBC *via* the classic culture method is in the range of weeks and such delays have a detrimental effect on treatment outcomes. Given that molecular testing is in the range of hours to 1 or 2 days its value in treating drug resistant TB cannot be overstated. When developing such tests, one wants to optimize each step so that tests are successful even when confronted with samples that have a low MTBC load or contain large amounts of host DNA. This could improve the performance of the popular rapid molecular tests, especially for samples with mycobacterial loads close to the limits of detection. Where optimizations could have a more significant impact is for tests based on targeted next generation sequencing (tNGS) which typically require higher quantities of DNA. This would be significant as tNGS can provide more comprehensive drug resistance profiles than the relatively limited resistance information provided by rapid tests. In this work we endeavor to optimize pre-treatment and extraction steps for molecular testing.

**Methods:**

We begin by choosing the best DNA extraction device by comparing the amount of DNA extracted by five commonly used devices from identical samples. Following this, the effect that decontamination and human DNA depletion have on extraction efficiency is explored.

**Results:**

The best results were achieved (i.e., the lowest C_t_ values) when neither decontamination nor human DNA depletion were used. As expected, in all tested scenarios the addition of decontamination to our workflow substantially reduced the yield of DNA extracted. This illustrates that the standard TB laboratory practice of applying decontamination, although being vital for culture-based testing, can negatively impact the performance of molecular testing. As a complement to the above experiments, we also considered the best *Mycobacterium tuberculosis* DNA storage method to optimize molecular testing carried out in the near- to medium-term. Comparing C_t_ values following three-month storage at 4 °C and at −20 °C and showed little difference between the two.

**Discussion:**

In summary, for molecular diagnostics aimed at mycobacteria this work highlights the importance of choosing the right DNA extraction device, indicates that decontamination causes significant loss of mycobacterial DNA, and shows that samples preserved for further molecular testing can be stored at 4 °C, just as well at −20 °C. Under our experimental settings, human DNA depletion gave no significant improvement in C_t_ values for the detection of MTBC.

## 1. Introduction

Prior to the coronavirus pandemic, TB was the leading cause of death from a single infectious agent throughout the globe. The WHO has invested considerable effort in developing a strategy aimed at ending global TB with early diagnosis combined with DST forming a key pillar of their strategy (World Health Organization, 2022). In recent years, molecular diagnostics has become vital in the fight against global TB and other infectious diseases (Soini and Musser, 2001; Zarei, 2017; Acharya et al., 2020). They provide a means of detecting disease-causing pathogens that is both fast and sensitive. The growing importance of such diagnostics techniques is illustrated not only by the frequency with which they are used, but also the increasing variety of methodologies that are being employed. Molecular diagnostics are of particular importance in allowing the rapid detection of slow-growing pathogens such as mycobacteria (Deggim-Messmer et al., 2016). These diagnostic techniques are at the forefront in the global battle against TB with methods such as targeted next generation sequencing (tNGS) allowing the creation of a comprehensive drug resistance profile within a short space of time. This means that for drug-resistant TB, the correct treatment can begin almost immediately instead of after weeks of delay. So molecular diagnostics are the bee’s knees, however, there remains one fly in the ointment, that of choosing the best protocol. While the methods used in the analysis of molecular targets are well established, the earlier pre-treatment and extraction steps warrant further optimization to establish the most successful protocols.

Choosing the best protocol for DNA extraction is far from clear-cut with the market offering numerous solutions. Traditionally DNA was isolated using manual phase separation methods (e.g., phenol/chloroform). These methods give high DNA yields with great reliability. However, these techniques are complicated, time consuming, and use toxic chemicals (Price et al., 2009). Over the years several commercially available DNA isolation solutions were developed which focused on obtaining DNA isolates that contain very few inhibitors. These are typically based on some sort of solid matrix that binds preferentially to nucleic acids followed by a series of cleaning steps to eliminate contaminants or inhibitors. While these extraction devices continue to improve in terms of their yields, purity, and ease of use, it is often unclear which of the many devices and protocols are best suited to extract DNA from a given organism. Extraction of DNA from mycobacteria is a somewhat unique case as their lipid-rich cell wall makes them difficult to lyse. Given the difficulty in lysing mycobacteria and the importance of maximizing DNA yield for successful molecular diagnostics, it is worth investigating which of the many available DNA extraction devices produces the highest DNA yield.

**Figure 1:**
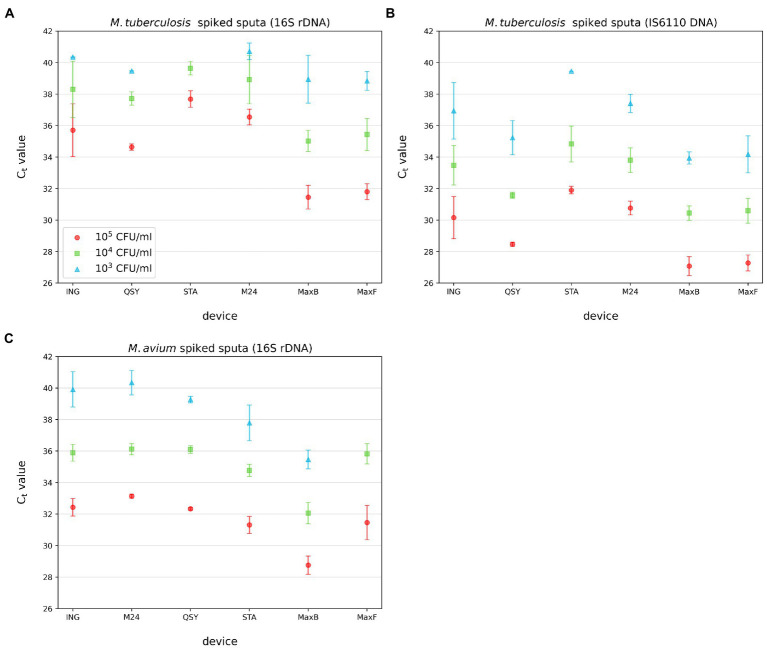
DNA yields for extraction with six device-kit combinations. Each plot **(A–C)** shows real-time PCR C_t_ values following extraction with each device-kit combination. Tests were carried out on sputa spiked with either *M. tuberculosis* **(A,B)** or *M. avi*um **(C)** at 3 concentrations ranging from 10^3^ to 10^5^ CFU/mL (colony-forming units/mL). The error bars give the standard deviation of the mean of experimental triplicates. Device-kit combinations are denoted: ING (ELITe InGenius [SP200 Kit]), QSY (Qiagen QIAsymphony [DSP Virus Pathogen Midi Kit]), STA (Hamilton Genomic STARlet [STARMag Universal Cartridge kit]), M24 (Roche MagNA Pure 24 [Total NA Isolation kit]), MaxB (Promega Maxwell 16 [RSC Blood DNA Kit]), and MaxF (Promega Maxwell 16 [RSC Pure Food Pathogen Kit]). Workflow: Sample => Decontamination (NALC-NaOH) => DNA isolation => qPCR.

In the preparation of mycobacteria for both culture and molecular amplification, decontamination of clinical specimens has long been standard practice (Kent and Kubica, 1985; Richter et al., 2019). Current laboratory workflows (see Figure 1; Dookie et al., 2022) for TB diagnostics employ decontamination as their first step, with the resulting sample subjected to a rapid test such as a real-time based test, or a line probe assay. These rapid tests can provide a result along with some resistance information in a matter of hours. While these rapid tests are being performed, part of the decontaminated sample proceeds to culture-based phenotypic DST which is much slower, taking up to 6 weeks, but is considered the gold standard in DST for TB. Another benefit of culture-based testing is that it produces isolates of sufficient quality to allow the application of molecular techniques, such as whole genome sequencing, that require larger amounts of target DNA. Given that it is standard in laboratory workflows to apply decontamination as a first step prior to diagnostic tests, it is worth investigating how to optimize its application to preserve sufficient DNA for molecular testing while at the same time maximizing the ability of mycobacteria to flourish in culture media. This study is focused solely on optimizing the DNA extraction workflow for molecular testing rather than attempting to find a treatment that simultaneously optimizes for both molecular testing and culture-based testing.

The main aim of decontamination is to remove most of the non-mycobacteria in the sample while not affecting the amount of viable mycobacteria. For culture-based analysis, decontamination is of vital importance as fast-growing commensal bacteria from clinical specimens can rapidly overgrow mycobacteria, which are typically extremely slow growing. Decontamination of sputum is performed using NALC (N-acetyl-l-cysteine) and NaOH where the aim is to preserve mycobacteria (Kubica et al., 1963). At sufficiently low concentrations NaOH breaks down the cell walls of most bacterial and human cells while leaving mycobacterial cells mostly uncompromised due to their lipid-rich cell wall. Prior treatment with the mucolytic agent NALC liquefies the sputum, and this allows the application of NaOH at lower concentrations thereby reducing its effect on mycobacteria while still lysing human cells and other bacteria. Even for treatment with such lower concentrations of NaOH it is still important to consider the quantity of mycobacteria that is lost as this could cause some molecular diagnostic techniques to produce false negatives on samples containing low mycobacterial loads. For this reason, the effect decontamination has on yields of mycobacterial DNA should be investigated, particularly when developing optimal workflows for molecular diagnostics on mycobacteria.

The amount of host and commensal DNA present in samples can have a significant effect on the success of molecular tests. Above we discussed decontamination which is aimed at removing both human and commensal DNA from our sample. For some molecular tests, for example real-time PCR or small-panel targeted sequencing, commensal DNA does not interfere with the assay if it is well designed. However, host DNA can still pose a problem due to the fact that in clinical specimens the overwhelming majority of DNA comes from the host and can end up being up to 99 % of the extracted DNA (Jurasz et al., 2021). This can lead to difficulty in amplifying target DNA, especially for samples with very low mycobacterial load. To address this problem human DNA can be selectively depleted by applying saponin to lyse non mycobacterial cells followed by DNase I which digests DNA post-lysis (Hasan et al., 2016; Charalampous et al., 2019). Once again it is not clear how much mycobacteria will be lost when applying these treatments and so it is worth exploring the effect they have on the final mycobacterial DNA yield when designing a workflow for molecular diagnostics on mycobacteria.

In culture-based tests for MTBC it is normal to place the sample in storage until there is a definitive answer from the test and it can be determined whether or not further testing is required. Due to the slow growth rate of mycobacteria this can involve storing the sample for months (usually at a temperature of −20 °C). However, the question remains, is storage at temperatures as low as −20 °C necessary or even optimal for short-term preservation of samples that may require further testing? In this work we look to shed light on this issue by comparing DNA yields following three-month storage at 4 °C and − 20 °C.

The quantity and quality of DNA available from the target pathogen is a key factor in successful molecular testing. The amount of host and commensal DNA is another factor that can affect the success of molecular tests. We believe that existing purification and extraction steps used in molecular diagnostic workflows aimed at mycobacteria can be optimized to improve DNA yields. This in turn would improve the reliability of molecular diagnostics, particularly on samples with low mycobacterial load. For example, it would increase the effectiveness of tNGS which is not as efficient on low quantities of DNA as some of the commonly used rapid tests such as those based on real-time PCR. In this work, the earlier stages in workflows aimed at mycobacteria are optimized: we identify which device, from five commonly used DNA extraction devices, gives the highest yield of mycobacterial DNA, and which pre-treatments involving the application or non-application of NALC-NaOH (for decontamination) and saponin-DNase I (for human DNA depletion) give the highest yield of *M. tuberculosis* DNA.

## 2. Materials and methods

### 2.1. Extraction of mycobacterial DNA

#### 2.1.1. Procedure used to produce mycobacterium spiked sputa

Colonies of *M. tuberculosis* (QK228) and *M. avium* (QK140) were homogenized with glass beads in phosphate buffered saline (PBS) to achieve 0.5 McFarland standard suspension of 1 × 10^7^ CFU/mL. *Mycobacterium* negative sputa were independently spiked with the McFarland cultures to achieve concentrations ranging from 1 × 10^5^ CFU/mL to 1 × 10^3^ CFU/mL for testing the nucleic extraction devices, from 1 × 10^5^ CFU/mL to 1 × 10^0^ CFU/mL for decontamination and human depletion experiments and ranging from 1 × 10^6^ CFU/mL to 10^1^ CFU/mL for storage experiments.

Working solutions (0.5 McFarland) used for making the spikes were assessed for cell count, on Middelbrook 7H10 agar after incubation for three weeks at 37 °C and were assessed for the number of copies of *M. tuberculosis* genomes using qPCR (16 s).

##### 2.1.1.1. Decontamination of samples with mycobacteria

The spiked sputa were aliquoted and then stored at −20 °C pending analysis. For each of the six device-protocol setups the sputa were decontaminated and inactivated in triplicate prior to DNA extraction with each setup. For decontamination, the sputa (1 mL) were subjected to an equal volume of 2 % NALC-NAOH solution, vortexed for 20s, and then incubated for 20 min at room temperature. Using sterile PBS, the sample volume was topped up to 45 mL and centrifuged at 4,480 × g for 15 min at 4 °C. The supernatant was decanted to leave a residual volume of 1 mL, and then inactivated.

##### 2.1.1.2. Human DNA depletion

Human DNA depletion was done using the QIAamp DNA Microbiome Kit (Qiagen, 51704). In brief 400 μL of Buffer AHL was added to 800 μL of sample. The solution was incubated for 30 min at room temperature, mixed every 5 min, and centrifuged at 10,000 × g for 10 min. The supernatant was removed, the pellet resuspended in 190 μL Buffer RDD and 2.5 μL Benzonase, and the solution was incubated for 30 min at 37 °C at 600 rpm in a thermo mixer. After incubation, 20 μL of Proteinase K was added and left for digestion for 30 min at 56 °C at 600 rpm in a thermo mixer. The tube was immediately centrifuged at slow speed and incubated at 80 °C for 30 min for pathogen deactivation. Pathogen lysis was achieved by adding 200 μL Buffer ATL (containing Reagent DX) to the sample. Samples were placed into a FastPrep-24 Instrument using a Pathogen Lysis Tube L and a velocity of 6.5 m/s was applied twice for 45 s each within a five-minute interval. The pathogen lysis tube was centrifuged at 10,000 × g for 1 min, the supernatant recovered, and 40 μL proteinase K was added. The lysate was incubated at 56 °C for 30 min at 600 rpm in a thermomixer. After incubation, 200 μL buffer APL2 was added and the lysate puls-vortexed for 30 s. Finally, the exposed DNA was isolated and purified using QIAamp UCP min spin columns according to manufacturer instructions. DNA was eluted in 50 μL AVE buffer and the DNA stored at −20 °C for further use.

##### 2.1.1.3. Inactivation of spiked sputum

Samples with mycobacteria were heat-inactivated at 95 °C for 20 min and vortexed at 800 rpm.

##### 2.1.1.4. Quantitative polymerase chain reaction (qPCR)

qPCR was carried out using TaqPath™ ProAmp™ Master Mix (Applied Biosystems, A30865), MTB assay with IS6110 primers and probe (TIB MolBiol, 53-0737-96) and RNaseP assay with RNaseP primers and probe (TIB MolBiol, 66-0907-96). qPCR was performed using a LightCycler^®^ 480 Instrument II, as per manufacturer’s instructions.

##### 2.1.1.5. Automated nucleic acid extraction from mycobacteria

The inactivated samples were processed according to the manufacturer’s recommendation using the six device-protocol setups described below. *M. tuberculosis* and NTM genome copy numbers were determined using the Seegene Anyplex real-time MTB/NTM detection kit according to the manufacturer’s recommendation (Seegene Inc., kit instructions for cat. TB7202Y).

##### 2.1.1.6. ELITe InGenius DNA extraction

Nucleic acids were extracted using ELITe InGenius (ELITechGroup, INT030-K) and STARMag 96×4 Universal Cartridge (Seegene, Catalog number). 200 μL of inactivated sputum was loaded onto the device. The nucleic acids were extracted according to manufacturer’s instructions (Elitech, ELITe InGeniusTM Device Manual) using the “generic” protocol and eluted with 100 μL of elution buffer provided by the supplier.

##### 2.1.1.7. Qiagen QIAsymphony DSP virus pathogen midi kit

Nucleic acids were extracted using QIAsymphony SP (Qiagen, 9001297) and QIAsymphony DSP Virus/Pathogen Kit (Qiagen, 937055). 400 μL of inactivated sputum was loaded onto the device. The nucleic acids were extracted according to manufacturer’s instructions (QIAGEN, kit instructions for cat. 937055) using Complex400_V4_DSP protocol and eluted with 60 μL of elution buffer provided by the supplier.

##### 2.1.1.8. Hamilton genomic STARlet STARMag university cartridge kit

Nucleic acids were extracted using Seegene STARlet (Seegene Inc., 173000-075) and STARMag 96×4 Universal Cartridge (Seegene, 744300.4.UC384). 500 μL of inactivated sputum was loaded onto the device. The nucleic acids were extracted according to manufacturer’s instructions (Seegene Inc., kit instructions for cat. TB7202Y) using the ‘one-step’ protocol and eluted with 60 μL of elution buffer provided by the supplier.

##### 2.1.1.9. Roche with MagNA pure 24 total NA isolation kit

Nucleic acids were extracted using Roche MagNA Pure 24 (Roche, 07290519001) and Roche MagNA Pure 24 Total NA Isolation Kit (Roche, 07658036001). 200 μL of inactivated sputum was mixed with 250 μL of lysis buffer and vortexed for 30 s. The resultant sample was incubated for 20 min at room temperature. Thereafter the sample was loaded into a cartridge along with 10 μL of IC. The nucleic acids were extracted according to manufacturer’s instructions and eluted with 50 μL of elution buffer provided by the supplier.

##### 2.1.1.10. Promega Maxwell RSC with blood DNA kit

Nucleic acids were extracted using Promega Maxwell RSC (Promega, AS 4500) and Maxwell RSC Blood DNA Kit (Promega, AS1400). 300 μL of inactivated sample was combined with 300 μL Lysis Buffer and 30 μL Proteinase K. The resultant mixture was incubated at 56 °C for 20 min while vortexing at 1,000 rpm. It was then loaded in the provided Maxwell Cartridge and DNA was extracted with 50 μL elution volume as per the manufacturer’s instructions.

##### 2.1.1.11. Promega Maxwell pure food pathogen kit

Nucleic acids were extracted using Promega Maxwell RSC (Promega, AS 4500) and Maxwell RSC Blood DNA Kit (Promega, AS1400). 500 μL of inactivated sample was combined with 300 μL of water and resultant mixture was incubated at 95 °C for 30 min. After inactivation, 200 μL of Lysis Buffer A was added and the resulting mixture was incubated at 56 °C for 4 min while vortexing at 1000 rpm. To each tube, 300 μL Lysis Buffer was added and mixed by vortexing for 10 s. The nucleic acids were extracted according to manufacturer’s instructions (Promega, instructions for AS4500) and eluted with 50 μL of elution buffer provided by the supplier.

### 2.2. Decontamination and human DNA depletion

Using spiked sputa with *M. tuberculosis* in concentrations ranging from 1 × 10^5^ to 1 × 10^0^ CFU/mL, four different DNA extraction protocols were compared by taking the final yield of *M. tuberculosis* genome copies and human genome copies. The following samples were measured for each DNA extraction route: human sputum containing no *Mycobacterium*; the pure culture of *M. tuberculosis* used for spiking, two negative controls (sterile NaCl and PBS), and *M. tuberculosis* spiked human sputum samples at the above-mentioned concentrations.

The following four DNA extraction routes were compared.

#### 2.2.1. Route 1: DNA extraction after decontamination and human DNA depletion

The samples were decontaminated and inactivated as described above. Human DNA was depleted using 250 μL buffer AHL, 190 μL Buffer RDD, 2.5 μL benzonase and 20 μL Proteinase K as described above, this was followed by incubation at 80 °C for 30 min. Cells were lysed using the pathogen lysis workflow with buffer ATL and the FastPrep-24 instrument as described above. The exposed DNA was isolated by QIAamp UCP min spin columns according to manufacturer instructions. DNA was eluted in 50 μL AVE buffer and the DNA stored at −20 °C for further use.

#### 2.2.2. Route 2: DNA extraction after decontamination without human DNA depletion

Samples were decontaminated and inactivated as described above. Cells were lysed using the pathogen lysis workflow with buffer ATL and the FastPrep-24 instrument as described above. The exposed DNA was isolated by QIAamp UCP min spin columns according to manufacturer instructions. DNA was eluted in 50 μL AVE buffer and the DNA stored at −20 °C for further use.

#### 2.2.3. Route 3: DNA extraction without decontamination but human DNA depletion

500 μL of each sample was depleted for host DNA using 250 μL buffer AHL, 190 μL Buffer RDD, 2.5 μL benzonase and 20 μL Proteinase K as described above, and this was followed by incubation at 80 °C for 30 min. Cells were lysed using the pathogen lysis workflow with buffer ATL and the FastPrep-24 instrument as described above. The exposed DNA was isolated by QIAamp UCP min spin columns according to manufacturer instructions. DNA was eluted in 50 μL AVE buffer and the DNA stored at −20 °C for further use.

#### 2.2.4. Route 4: DNA extraction without decontamination and human DNA depletion

For sample inactivation, 500 μL of each sample was incubated at 80 °C for 30 min and the tube was briefly centrifuged. Following this, cells were lysed using the pathogen lysis workflow with buffer ATL and the FastPrep-24 instrument as described above. The exposed DNA was isolated by QIAamp UCP min spin columns according to manufacturer instructions. DNA was eluted in 50 μL AVE buffer and the DNA stored at −20 °C for further use.

### 2.3. Storage of *Mycobacterium tuberculosis*

*Mycobacterium tuberculosis* spiked sputum samples were prepared in concentrations ranging from 10^6^ to 10^1^ CFU/mL as described above. For each of these concentrations, 500 μL were stored in duplicates at 4 °C and −20 °C. For each storage temperature, a *Mycobacterium tuberculosis* (*M. tuberculosis*) culture at 0.5 McFarland, a sputum prior to spiking (*M. tuberculosis* negative), and a PBS solution was stored. Prior to storage, *M. tuberculosis* DNA was extracted from each spiked sputa in duplicates (time zero) using Promega Maxwell RSC Blood Kit. *M. tuberculosis* DNA yield was measured by qPCR in duplicates. The same procedure was repeated with stored sputa after 3 months at 4 °C and at −20 °C.

## 3. Results

For testing the performance of different extraction devices, Mycobacterial loads between 10^3^ to 10^5^ CFU/mL were chosen as these are typical for patients with symptomatic infections. For our decontamination and human depletion experiments improved DNA yields were expected when decontamination was dropped (Affolabi et al., 2012; Mtafya et al., 2019; Srivastava et al., 2020) and so mycobacterial loads as low as 10^0^ CFU/mL were tested. For the storage experiment a deterioration in performance was expected following 3 months storage and so mycobacterial loads between 10^1^ CFU/mL to 10^6^ CFU/mL were tested.

### 3.1. Extraction of mycobacterial DNA

Our institution uses three DNA isolation devices for routine diagnostics of suspected tuberculosis cases: the ELITe InGenius, the Qiagen QIAsymphony, and the Hamilton Genomic STARlet. Comparing these three devices we observed considerable variability in C_t_ values during real-time PCR analysis of samples containing *M. tuberculosis*. For this reason, we implemented the extraction experiments detailed in the Materials and Methods section to investigate the performance of the three devices in use at our institution as well as two further devices that are widely used in diagnostic laboratories in Europe (the Promega Maxwell 16 and the Roche MagNA Pure 24). We tested two commonly used kits on the Promega Maxwell and one on each of the other devices. Tests were carried out on sputa spiked with *M. tuberculosis* to emulate TB infections, and on sputa spiked with *M. avium* to emulate nontuberculous mycobacterial infections.

The input sample volumes were variable between devices as they are limited by the design of each device. We used the maximum sample volume recommended for each device to optimize the performance of each device. Input volumes ranged from 200 μL to 500 μL, which should give a loss or gain of around 1 C_t_ value, all else being equal.

From our experiments (see Figure 1), we noticed that the extraction efficiency for mycobacterial DNA was highly variable between the different setups. Promega was by far the best extraction device in our experiments with both Promega setups achieving the lowest C_t_ values in all experiments. Of the two Promega setups the RSC Blood DNA Kit was the overall winner, with slightly lower C_t_ values when detecting *M. tuberculosis* and significantly lower C_t_ values for *M. avium*. The Seegene setup gives the highest C_t_ values. All this translates to a low mycobacterial DNA yield for the Seegene setup versus a high DNA yield for the Promega setup. The yields from the other systems were between Seegene and Promega Blood Kit.

The best performing setup, the Promega blood DNA kit, was compared *via t* test to each of the other setups over equivalent concentrations. The Promega blood kit gave a decrease in C_t_ values with a statistical significance of *p* < 0.05 compared to the ELITe InGenius setup, a statistical significance of *p* < 0.025 compared to the Hamilton Genomic setup, and excluding the point at IS6110 at 10^3^ CFU/mL the Promega blood kit gave lower C_t_ values with a statistical significance of *p* < 0.01 compared to both the Roche and Qiagen QIAsymphony setups. When using the Promega the decrease in C_t_ values with blood DNA kit compared to the Pure Food Pathogen kit was not statistically significant for *M. tuberculosis* (*p* > 0.05) but was significant for *M. Avium* (*p* < 0.01).

### 3.2. Decontamination and human DNA depletion

We tested four treatments on healthy sputa spiked with *M. tuberculosis* at concentrations ranging from 10^5^ to 10^0^ CFU/mL:

- Decontamination and human DNA depletion
- Decontamination and no human DNA depletion
- No decontamination and no human DNA depletion
- No decontamination with human DNA depletion

Post treatment DNA was extracted using Promega Maxwell RSC Blood DNA Kit which we had previously identified as giving the most efficient DNA extraction. DNA yield was estimated using real-time PCRs for 16s rDNA and IS6110 for *M. tuberculosis*, and RNaseP for *H. sapiens*.

**Figure 2:**
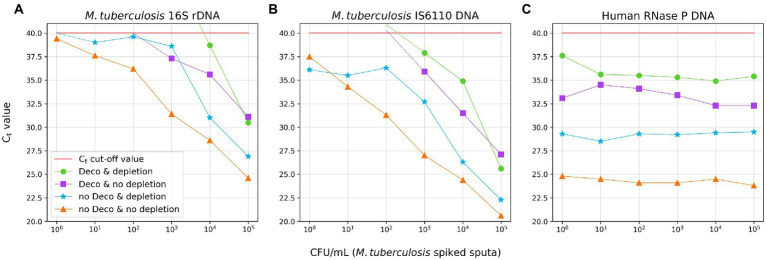
DNA yields for decontamination and human DNA depletion experiment. Each plot **(A–C)** shows real-time PCR C_t_ values following the four possible treatment options involving the application or non-application of decontamination (NALC-NaOH) and depletion (saponin and DNase I). Tests were carried out on sputa spiked with *M. tuberculosis* at 6 concentrations ranging from 10^0^ to 10^5^ CFU/mL (colony-forming units/mL). Plot gives the mean of biological duplicates.

From Figures 2A,B the best yields of mycobacterial DNA were obtained with no treatment at all (no decontamination and no depletion). We found that decontamination greatly reduced yields of mycobacterial DNA. The addition of decontamination increased C_t_ values by an average of 6.4 for samples with concentrations of 10^5^ to 10^3^ CFU/mL, and for samples with 10^2^ CFU/mL or lower its addition resulted in a complete loss of detection with real-time PCR.

In contrast to decontamination, human DNA depletion showed encouraging results. Although it led to an average decrease of 2.3 in C_t_ value for *M. tuberculosis* DNA (Figures 2A,B), it also led to an average decrease of 4.9 for human DNA in the absence of decontamination and 2.4 in the presence of decontamination (Figure 2C). For a simple assay like real-time PCR human DNA depletion may not be of much benefit but in highly multiplexed PCR, tNGS, or metagenomic assays such preferential reduction in human DNA could offer significant improvement.

When decontamination and depletion are applied together, the C_t_ values increase rapidly *as the* concentration of *M. tuberculosis* decreases (green curve Figures 2A,B). In fact, the performance deteriorates so rapidly that only the two highest concentrations are within the limit of detection. This is not the case for the other treatment combinations tested, their C_t_ values increase at a slower rate as the concentration of *M. tuberculosis* decreases, and for treatment with depletion only, the rate of increase slows as illustrated by a flattening of the blue curve at lower concentrations (Figures 2A,B).

The increase in C_t_ value for *M. tuberculosis* resulting from the application of decontamination was statistically significant over all comparable points (*t* test, *p* < 0.05 at each concentration for 16S and IS6110), whereas the increase in C_t_ value for *M. tuberculosis* resulting from the application of saponin was not statistically significant for all comparable points (*p* > 0.05 for half the points from 16S and IS6110).

The increase in C_t_ value for RNaseP resulting from the application of decontamination was statistically significant over all comparable points (*t* test, *p* < 0.05 at each concentration). When decontamination is used the increase in C_t_ value for RNaseP that results from the addition of saponin was not statistically significant for most concentrations (*p* > 0.05 for all except 10^4^ CFU/mL). When decontamination is not used the increase in C_t_ value for RNaseP that results from addition of saponin was statistically significant at all but the lowest concentration (*p* < 0.01 for10^5^ to 10^1^ CFU/mL, *p* > 0.05 for 10^0^ CFU/mL).

### 3.3. Storage of *Mycobacterium tuberculosis*

Samples containing or suspected to contain mycobacteria are routinely stored at 2–8 °C for short term storage and −20 °C for long term storage. Occasionally further testing is required, or the primary sample needs to be retested due to contamination or other experimental failure. As part of our efforts to optimize diagnostic workflows for mycobacteria, we wanted to understand the effect storage temperature has on DNA yield and so we compared C_t_ values following three-month storage at 4 °C and −20 °C.

**Figure 3:**
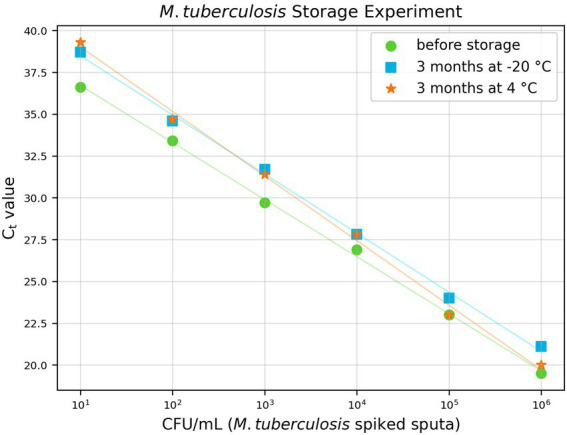
Real-time PCR C_t_ values before storage and following 3-month storage at 4°C and at −20°C. Tests carried out on sputa spiked with *M. tuberculosis* at 6 concentrations ranging from 10^1^ to 10^5^ CFU/mL (colony-forming units/mL). Plot gives the mean of experimental duplicates. Workflow: Sample => DNA isolation => qPCR.

From Figure 3 we see that there is little difference in C_t_ value following three-month storage at 4 °C and at −20 °C. Three-month storage at either temperature does not cause much loss of mycobacterial DNA when compared with immediate usage. As the concentration decreases there is a slight increase in C_t_ values when comparing three-month storage to immediate usage: at the highest concentrations 3-month storage causes only small losses in mycobacterial DNA and at the lowest concentration we see some increase in the amount lost (an increase of 2.7 in C_t_ value).

Analysis of the variance (ANOVA) on the three sets (before storage, 3 months at 4 °C, 3 months at −20 °C) found that the difference in C_t_ values at concentrations 10^6^ CFU/mL was statistically significant (*p* < 0.05), and not statistically significant at concentrations 10^5^ to 10^1^ CFU/mL (*p* > 0.05).

## 4. Discussion

The aim of this study was to optimize the preparation and treatment of clinical specimens to improve mycobacterial DNA yield for molecular testing. This is particularly important for tests that are unreliable at low mycobacterial loads, as is the case for most patient specimens (Najjingo et al., 2019). Improving the sensitivity of such tests should be made a priority as they can address the shortcomings of other more reliable diagnostic tests. Many of these more reliable tests employ amplification techniques that achieve high sensitivity when started on tiny quantities of material. Assays that are based on amplifying target material by culture, where single cells grow into colonies, offer a means of carrying out highly sensitive DST for multiple drugs simultaneously. Unfortunately, it takes time for cells to multiply sufficiently, and for slow growing bacteria, such as mycobacteria, susceptibility testing by culture can take weeks. This means that if we rely solely on culture for DST the optimal treatment regimen can be delayed by up to two months (Deggim-Messmer et al., 2016). Assays based on molecular diagnostic techniques work by amplifying targeted regions in the genome to give millions of copies of each target that can then be detected/sequenced. Many of these assays are designed to amplify only a small number of targets (e.g., real-time PCR, isothermal amplification), and so they do not cover enough drugs to identify the best treatment regimen for strains that are resistant to multiple drugs [e.g., MDR-TB, XDR-TB, and TDR-TB (World Health Organization, 2020)].

Next, generation molecular technologies can overcome the problems mentioned in the previous paragraph: they can amplify a large number of targets to produce comprehensive drug resistance profiles and can do so without the delay associated with bacterial culture. However, amplifying multiple targets poses its own challenges as it requires having multiple reactions in the same environment (one for each target). This places restrictions on the primer pairs we can choose to drive the amplification reactions. We need to ensure that there are no unwanted interactions between all primer pairs and that all primers anneal within the same temperature range. These and other restrictions result in lower amplification efficiency and so we need starting material that is orders of magnitude higher when compared to the techniques mentioned above. Since the DNA in clinical specimens that can be used for diagnosis is limited and often cannot be increased without culture-based methods, it is worthwhile spending time optimizing the process so that we lose as little DNA as possible. When designing a workflow for molecular testing it is also important to consider the negative effect caused by the presence of DNA from both the host and commensal bacteria. However, it is not simply a matter of applying treatments to remove unwanted DNA as any treatment could also decrease the quantity and quality of DNA from the target pathogen. So, in the absence of definitive evidence, it is worthwhile to find out whether our molecular diagnostic test performs better with or without treatments designed to remove the DNA of non-target organisms.

One of the most widely used treatments that facilitates the removal of unwanted DNA in workflows that test for mycobacteria is decontamination with NALC-NaOH. This treatment has long been part of the recommended workflows of commercially available molecular diagnostics test for TB including Xpert MTB/RIF (Cepheid, 2020), Xpert MTB/XDR (Cepheid, 2021), BD MAX (Becton, Dickinson and Company, 2020), and the Amplicor and E-MTD tests (Soini and Musser, 2001). The basis of this treatment is that the lipid-rich cell walls of mycobacteria provide a greater resistance to NaOH induced lysis than other cells. However, it has been noted (Pfyffer, 2015) that mycobacteria are only slightly more resistant to treatment with NaOH than the cells we wish to lyse, and there have been studies demonstrating the negative effects of decontamination. In molecular tests for *Mycobacterium ulcerans* all three setups (single-run PCR, nested PCR and real-time PCR) performed better on untreated tissue samples compared to those treated with NALC-NaOH (Affolabi et al., 2012). On serial dilutions of stock *M. tuberculosis,* a reduction of 4.36 ± 0.13 log_10_ CFU/mL was observed in cultures following treatment with NALC-NaOH, which also resulted in an increase in the limit of detection from 0.42 log_10_ CFU/mL to 1.47 log_10_ CFU/mL (Srivastava et al., 2020). With the molecular bacterial load assay, NALC-NaOH treatment was found to raise the limit of detection by 0.72 ± 0.08 log_10_ CFU/mL for *M. tuberculosis* pure culture, and by 0.65 ± 0.17 log_10_ CFU/mL for clinical sputum samples (Mtafya et al., 2019).

While the literature gives evidence that treatment with NALC-NaOH may result in losses of mycobacterial DNA, most of the recommendations from manufactures of commercial assays and the official guidelines advise using NALC-NaOH decontamination. And so, we were somewhat surprised that decontamination applied to sputum samples caused such a significant reduction in DNA yields of *M. tuberculosis* (Figures 2A,B). The addition of decontamination to each workflow caused an average increase of 6.5 in the C_t_ value. From our experiments decontamination causes too much loss of mycobacterial DNA to be of benefit for real-time PCR. Given the large increase in C_t_ value with the introduction of decontamination it is quite likely that it would have a negative effect on other molecular diagnostics techniques for mycobacteria, and as we have seen that there is evidence for this in the literature (Affolabi et al., 2012; Mtafya et al., 2019; Srivastava et al., 2020).

The vast quantities of human DNA contained in clinical samples can result in up to 99 % of extracted DNA coming from the host (Jurasz et al., 2021), and this hinders the efficiency of downstream molecular testing for pathogens. Human DNA can be selectively depleted by adding saponin to lyse human cells and then DNase I to digest the DNA released by cell lysis. Saponin has traditionally been used in hematology labs to lyse erythrocytes without harming pathogens, and more recently to deplete the amount of human DNA prior to sequencing (Faria et al., 2015). Saponin-DNase I treatment is becoming a popular method of depleting human DNA in other clinical settings. In cerebrospinal fluid and nasopharyngeal aspirate specimens spiked with bacterial and viral pathogens the amount of pathogen specific NGS reads were compared following treatment with and without saponin-DNase I (Hasan et al., 2016). The treatment with saponin-DNase gave a 20- to 650-fold increase in the ratio of microbial reads to human reads, with a significant reduction in the number of human reads and improved sensitivity in pathogen detection. Saponin based human DNA depletion on respiratory samples was shown to remove up to 99.9 % of host DNA (Charalampous et al., 2019). Also, excluding *S. pneumoniae*, depletion on sputa spiked with common respiratory pathogens resulted in little difference in average C_t_ values (average difference < 1) for real-time PCR detection of pathogens.

So, the literature indicates that depletion with saponin can improve downstream molecular testing. Our results are not so clear-cut, we found that the addition of human DNA depletion to the workflow reduced the average yield of *M. tuberculosis* DNA with an average increase in C_t_ value of 2.3 for real-time PCR. However, it is likely that human DNA depletion could be useful in other settings as the increase in C_t_ value was relatively small, and it significantly reduced the amount of human DNA. It is also worth noticing that at lower *M. tuberculosis* concentrations the addition of human DNA depletion to the workflow resulted in only a slight increase in C_t_ values and actually lowered the C_t_ value in one instance. This may indicate that human DNA depletion could benefit molecular diagnostics that perform poorly on samples containing low *M. tuberculosis* loads. For such techniques, the modest increase in C_t_ values observed at higher *M. tuberculosis* concentrations could be a worthwhile trade-off for improved performance at lower concentrations as C_t_ values at higher concentrations should remain well within the limit of detection when the increase in C_t_ value is relatively small. Human DNA depletion may be of little benefit in molecular diagnostics with few targets but could be beneficial for highly multiplex assays.

Mycobacteria are notorious for being difficult to lyse using off-the-shelf commercial methods due to their mycolic acid-containing cell wall and their ability to grow in biofilms. We noticed considerable differences in DNA isolation efficiency between the evaluated automated nucleic acid systems. In our experiments the Promega setup using the RSC Blood DNA Kit^1^ gave the highest yields of mycobacterial DNA (Figure 1). The unique difficulties in lysing mycobacteria and the variability we encountered between the various extraction devices would indicate that it is worth optimizing the standard protocols installed on commercial extraction devices for mycobacteria. Given the current variability in performance, it is critical for diagnostic labs to evaluate the efficiency of extraction devices as poor DNA isolation could lead to false negative results of genus-specific assays on samples that have low mycobacterial load.

Storage is another important factor to consider for successful diagnostic testing, as it is often necessary to place patient samples in short-term storage. This can happen because the resources to carry out testing are not immediately available when samples arrive, and even if testing can be carried out immediately it is good practice to maintain samples in case further testing is required. For this reason, identifying which short-term storage methods best maintain samples for testing is an important consideration when developing diagnostic workflows. We used real-time PCR to compare DNA yields following three-month storage at 4 °C and −20 °C. On the plus side, storage at 4 °C is cheaper, less equipment intensive, and will not kill mycobacteria. However, 4 °C might not stop other microorganisms growing, and it was not clear at the outset if this would negatively affect DNA yield. This concern turned out to be somewhat unfounded as there was little difference in C_t_ values following three-month storage at both temperatures (Figure 3). When compared with C_t_ values obtained from immediate testing, 3-month storage showed only a minor increase in C_t_ values at higher mycobacterial loads, with the increase becoming slightly more pronounced at lower concentrations. So, while the convention is to store samples at −20 °C, there seems to be no significant downside to short-term storage at 4 °C when the aim is to preserve samples for mycobacteria molecular diagnostics.

While this study considered key factors in the design of molecular diagnostic workflows, it still has its limitations. We used healthy sputa spiked with mycobacteria as it is extremely difficult to get sufficient quantities of infected patient samples to allow comparison of multiple parameters. However, we would expect similar findings for clinical specimens, and this is a topic for future investigation. In this study we evaluated real-time PCR only, which is one of the best molecular techniques for detecting very low amounts of pathogen DNA. In future studies it would be of interest to investigate how other molecular assays perform when following the steps found to be optimal in this work. This would be of particular interest for assays that usually need higher amounts of target DNA such as highly multiplex PCR, targeted sequencing, DNA hybridization or metagenomic assays. Given their need for higher mycobacterial loads such molecular techniques could, as we found for real-time PCR, benefit from excluding decontamination as well as choosing the device best suited to extract mycobacterial DNA. It is worth noting that the steps found to be optimal in this study were also used to isolate DNA for use by a 96-target multiplex PCR assay^2^. In fact, with the steps identified here, this assay was found to be effective on samples in the range of very low *M. tuberculosis* loads (very low as per the GeneXpert semi-quantitative classification (Blakemore et al., 2011)).

Another item to be addressed in the future is to determine how effective decontamination is at reducing the DNA of commensal bacteria and other non-mycobacterial DNA, as this may help us identify situations where decontamination could be useful. In this study any contaminant flora removed by the NALC-NaOH treatment did not outweigh the negative impact it had on mycobacterial DNA yields. However, such contaminant flora could have a greater effect on highly multiplexed tNGS and some of the other molecular techniques mentioned above and so its potential impact cannot be ignored. The effects of contaminant flora can be somewhat mitigated by careful primer design paying particular attention to off-target effects, but this is a design issue for optimizing the downstream workflow not the pretreatment and extraction steps.

The evolution of workflows for molecular testing has borrowed much from culture-based DST. Sample preparation and treatment prior to molecular testing frequently follows steps traditional in culture-based workflows. However, one should be wary of accepting steps that have been tried and tested for culture as the default for molecular diagnostics. It is reasonable to assume that any form of pre-treatment has the potential to reduce the quantity of pathogen DNA that can be extracted and thus have a negative effect on molecular testing, particularly at low pathogen loads. This was a major motivating factor in our decision to test whether or not decontamination and human DNA depletion had a significant effect on the quantity of mycobacterial DNA obtained from sputa. As it turned out, the reduction in DNA yield was much larger than we anticipated. Our results show that decontamination significantly had a detrimental effect on mycobacterial DNA yield, and that while human DNA depletion also reduced DNA yield, the effect was much less pronounced. The huge reduction in yield caused by decontamination adds to previous evidence that argues against its inclusion in molecular diagnostic workflows targeting mycobacteria. Such arguments against the inclusion of human DNA depletion are tempered by the fact that the reduction in DNA yield was much less, and by the fact that at lower mycobacterial concentrations the reduction was negligible. It is possible that when the amount of mycobacterial DNA is very low, and the amount of human DNA is many orders of magnitude higher, human depletion could improve the performance of some molecular tests. In our investigation involving commonly used extraction devices we found considerable variability in mycobacterial DNA yields indicating that the choice of extraction device can have a significant effect on the reliability of molecular testing.

In conclusion, for optimal diagnostics of mycobacteria, avoid decontamination, choose the right automated nucleic acid extraction process, and for complex assays remove human DNA if necessary. Mycobacterial DNA can be served cold or frozen.

## Data availability statement

The original contributions presented in the study are included in the article/Supplementary material, further inquiries can be directed to the corresponding author.

## Author contributions

PP, PK, and SD designed the study. PP and KR carried out the lab work. TN, PP, and PG wrote the manuscript. PP, PG, and TN carried out the data analysis. PP, PK, TN, PB, and TB reviewed and edited the manuscript. All authors contributed to the article and approved the submitted version.

## Funding

This study was funded by Innosuisse - Swiss Innovation Agency (grant number 36198.1 IP-LS) and open access funding by University of Bern.

## Conflict of interest

This work was funded by an Innosuisse project whose industrial partner is Clemedi AG, a Medtech company that develops *in vitro* molecular diagnostic solutions. SD, PK, TB, and PP hold shares in the company, and TB is on the board of directors. SD is Clemedi CEO, PP is Clemedi CSO, TN and PK are part of Clemedi’s R&D team, PG worked for Clemedi in the past.

The remaining authors declare that the research was conducted in the absence of any commercial or financial relationships that could be construed as a potential conflict of interest.

## Publisher’s note

All claims expressed in this article are solely those of the authors and do not necessarily represent those of their affiliated organizations, or those of the publisher, the editors and the reviewers. Any product that may be evaluated in this article, or claim that may be made by its manufacturer, is not guaranteed or endorsed by the publisher.

## Footnotes

^1^It is worth noting that this setup (the Promega Maxwell with RSC Blood DNA Kit) compares favorably to other devices in terms of cost per sample and setup time. For a comparison between the devices tested in this work see the Supplementary Table 1.

^2^This 96-target assay is known as Tuberculini, a recently certified molecular diagnostic test for TB that gives a 12-drug resistance profile. The details of these results are not reported in this work but will be published in the near future.

## Abbreviations

TB: Tuberculosis
*M. tuberculosis*: *Mycobacterium tuberculosis*
WHO: World Health Organization
DST: drug susceptibility testing
C_t_: cycle threshold
NALC: N-acetyl-L-cysteine
PBS: phosphate buffered saline
*M. avium*: *Mycobacterium avium*
*M. abscessus*: *Mycobacterium abscessus*
MDR-TB: multidrug-resistant tuberculosis
XDR-TB: extensively drug-resistant tuberculosis
TDR-TB: totally drug-resistant tuberculosis
NGS: next-generation sequencing
*S. pneumoniae*: *Streptococcus pneumoniae*
